# Butterfly Wing Color Pattern Modification Inducers May Act on Chitin in the Apical Extracellular Site: Implications in Morphogenic Signals for Color Pattern Determination

**DOI:** 10.3390/biology11111620

**Published:** 2022-11-06

**Authors:** Joji M. Otaki, Yugo Nakazato

**Affiliations:** The BCPH Unit of Molecular Physiology, Department of Chemistry, Biology and Marine Science, Faculty of Science, University of the Ryukyus, Okinawa 903-0213, Japan

**Keywords:** butterfly wing, color pattern formation, color pattern modification, chitin, cuticle, extracellular matrix, eyespot, organizer, morphogen, Wnt signaling

## Abstract

**Simple Summary:**

Butterfly wing color patterns are modified by various treatments, but their mechanisms of action have been enigmatic. We hypothesized that these modification-inducing treatments act on the pupal cuticle or extracellular matrix (ECM). Mechanical load tests revealed that pupae treated with cold shock or chemical inducers were significantly less rigid, suggesting that these treatments made cuticle formation less efficient. A known inhibitor of cuticle formation that binds to chitin, FB28 (fluorescent brightener 28), was discovered to efficiently induce modifications. Fluorescent signals from FB28 were observed in live pupae in vivo from the apical extracellular side and were concentrated at the pupal cuticle focal spots immediately above the eyespot organizing centers. Taken together, various modification-inducing treatments likely act extracellularly on chitin or its related polysaccharides to inhibit pupal cuticle formation or ECM function, which probably causes retardation of morphogenic signals.

**Abstract:**

Butterfly wing color patterns are modified by various treatments, such as temperature shock, injection of chemical inducers, and covering materials on pupal wing tissue. Their mechanisms of action have been enigmatic. Here, we investigated the mechanisms of color pattern modifications using the blue pansy butterfly *Junonia orithya*. We hypothesized that these modification-inducing treatments act on the pupal cuticle or extracellular matrix (ECM). Mechanical load tests revealed that pupae treated with cold shock or chemical inducers were significantly less rigid, suggesting that these treatments made cuticle formation less efficient. A known chitin inhibitor, FB28 (fluorescent brightener 28), was discovered to efficiently induce modifications. Taking advantage of its fluorescent character, fluorescent signals from FB28 were observed in live pupae in vivo from the apical extracellular side and were concentrated at the pupal cuticle focal spots immediately above the eyespot organizing centers. It was shown that chemical modification inducers and covering materials worked additively. Taken together, various modification-inducing treatments likely act extracellularly on chitin or other polysaccharides to inhibit pupal cuticle formation or ECM function, which probably causes retardation of morphogenic signals. It is likely that an interactive ECM is required for morphogenic signals for color pattern determination to travel long distances.

## 1. Introduction

Among the diverse body color patterns in animals, butterfly color patterns are probably unique in that their components, called color pattern elements, are considered anatomical entities and are different from the color patterns of other animals, such as giraffes and zebras [1,2]. A standard positioning of the color pattern elements is known as the nymphalid groundplan, and this groundplan is modified developmentally and evolutionarily in size, shape, and position to produce a new color pattern for a new species [1,2,3,4,5,6]. This pattern transformation on a two-dimensional space (i.e., on a wing surface) is considered simpler than most other three-dimensional developmental and evolutionary changes. Yet, the butterfly wing color pattern system likely retains the essence of morphogenesis in biological development [6].

The development of these color pattern elements has been studied genetically at the molecular level, identifying many genes that are involved in this process [7,8,9,10,11,12,13,14,15,16,17,18,19,20,21,22,23,24,25,26,27,28]. Representative genes are probably *Distal-less* [7,8,10,12,14,15,16,22,26] and *Wnt* genes [10,12,13,18,19]. Although genetically controlled, the development of color pattern elements can be manipulated by physiological treatments. It has been known that color patterns can be modified by cold shock [1,29], but it was not until 1998 that chemical modification inducers were discovered [30]. Through injections of chemicals into pupae, sodium tungstate has been identified as a potent modification inducer [30]. Tungstate-induced modifications are very similar, if not identical, to those induced by cold shock; parafocal elements (PFEs) are thickened and dislocated toward the corresponding eyespots, and the eyespots themselves are often compromised [30]. This type of modification is called temperature-shock-type (TS-type) modification [31].

It is important to stress that with these TS-type modification inducers, color pattern elements are modified in terms of size, shape, and position [31]. This fact implies that the morphogenic signaling system for color pattern determination is directly modified [31]. Furthermore, TS-type modifications are different from those based on the general stress response induced by thapsigargin and other chemicals and from those induced by ecdysteroids [32]. In this sense, the importance of investigating the mechanisms of TS-type modifications cannot be overemphasized in developmental biology.

Other TS-type modification inducers were then discovered in 2003 [33] and 2005 [34]. The inducer discovered by Umebachi and Osanai (2003) [34] is an acid carboxypeptidase, which is structurally very different from tungstate. The inducers discovered by Serfas and Carroll (2005) [34] are polysaccharides such as heparin, which are also structurally very different from tungstate. Because of their high molecular weights, it has been speculated that these polysaccharide inducers act extracellularly [34]. Heparin is considered to enhance Wnt signaling [13,19]. Among the modification-inducing polysaccharides, a unique inducer, dextran sulfate, appears to act in an opposite way to other TS-type inducers, including tungstate and heparin; PFEs are dislocated away from the corresponding eyespots and are often enhanced (but occasionally thinned). This modification pattern is somewhat similar to the “reversed” TS type often induced by heat shock treatments [35]. That is, the distance between a PFE and its corresponding eyespot is larger in heat-shocked individuals than in no-treatment or cold-shocked individuals, as if the morphogenic signal propagation was accelerated. Heat shock treatment produces variable modifications compared with cold shock treatment [35,36].

Upon the discovery of modification inducers, Otaki (1998) [30] discussed the physiological mechanisms of cold shock-induced modifications, speculating that cold shock induces cold shock hormone (CSH) to act like tungstate and that cold shock simultaneously acts directly on the wing tissue. These speculations are based on the following reasons. Modification-inducing properties can be transferable via hemolymph transfusion, and all individuals show symmetric modifications in the right and left wings [30]. Serfas and Carrol (2005) [34] also speculated for humoral factors because asymmetric modifications were not obtained in their treatments. Later, a series of experiments demonstrated that modification-inducing properties can be transferable via parabiosis, that modifications are induced without the head and a part of the thorax, and that transplantation of the tracheae and their associated tissues can transfer modification-inducing properties [37]. However, these results do not exclude the possibility that a local factor may also be responsible for modifications in addition to a humoral factor. To support a local factor, asymmetric modifications have been achieved by local application of tungstate or cold shock [38,39].

Surprisingly, TS-type modifications can be locally achieved by hydrophobic covering materials on the dorsal surface of the hindwing [40,41]. This discovery indicates that the site of action of modifications is indeed extracellular. Interactions between the cuticular extracellular matrix (ECM) and wing epithelial cells appear to determine the morphological features of PFEs and eyespots. In other words, morphogenic signals for color pattern elements may be regulated by extracellular interactions. This hypothesis may be called the “pupal cuticle hypothesis” in the present study. If this hypothesis is correct, we should be able to identify novel TS-type modification inducers from known inhibitors of the cuticle formation process, including secretion, chitin assembly, sclerotization, or other steps.

In this study, we used the blue pansy butterfly *Junonia orithya*, as has been used in previous studies. This species has large eyespots. To examine the pupal cuticle hypothesis, we performed a mechanical load test to quantify the effects of modification-inducing treatments on the pupal exoskeleton. We also performed injections of several candidate inhibitors for cuticle or chitin. We discovered that one of them, fluorescent brightener 28 (FB28), was an excellent TS-type modification inducer. Taking advantage of the fact that FB28 is fluorescent, we investigated the site of action of FB28. We propose that other TS-type modification inducers and treatments, such as cold shock, tungstate, heparin, and hydrophobic covering materials, may also act similarly at the apical extracellular site by inhibiting cuticle formation and thereby inhibiting morphogenic signal propagation, resulting in compromised eyespots and PFEs. These results were discussed in light of conventional molecular (chemical) morphogens such as Wnt as well as unconventional mechanical (physical) morphogens.

## 2. Materials and Methods

### 2.1. Butterflies

Adult females of the blue pansy butterfly, *J. orithya*, were field-collected on the Nishihara Campus of the University of the Ryukyus. They were confined in a glass tank (300 × 300 × 300 mm) together with the host plant (*Phyla nodiflora* and/or *Plantago asiatica*) at ambient temperatures to collect embryos. Larvae were reared in plastic containers using host plant leaves until pupation. Pupae were subjected to various experimental treatments.

**Figure 1:**
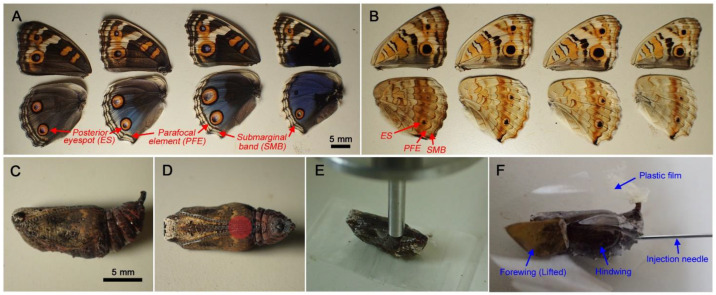
Wing color patterns and experimental procedures. (**A**) Dorsal wings of three females (left) and one male. The posterior eyespot (ES), distal parafocal element (PFE), and submarginal band (SMB) are indicated. (**B**) Ventral hindwings of individuals shown in A. ES, PFE, and SMB are indicated. (**C**) Side view of a pupa. This pupa is immediately before eclosion. Forewing color patterns are seen through the pupal cuticle. (**D**) Ventral view of a pupa. The position of a gauge probe is shown by a red circle. (**E**) A pupa with a gauge probe for the mechanical load test. (**F**) A pupa with a forewing lift configuration with an injection needle. Lifted wing surfaces are covered with a piece of transparent plastic film.

The wing color patterns of this butterfly were variable on the dorsal side of females (less variable in males) in terms of background coloration and eyespot size (Figure 1A). The ventral side contained smaller eyespots and other elements (Figure 1B). For color pattern modifications induced by cold shock, chemical injections, and covering materials, we focused on eyespots (ESs) and distal parafocal elements (PFEs) (and submarginal bands (SMBs) to a lesser extent), because these elements were most sensitive to treatments. Pupae have distinct pupal cuticle focal spots and marks (sensu Otaki et al., 2005 [42]) on the dorsal surface of the pupal forewing (Figure 1C). Pupal cuticle focal “spots” are cuticular focal bumps or dips on the surface located immediately above the eyespot organizing center in the wing epithelium [42]. They are often surrounded by dark circular “marks” in *Junonia* butterflies [42]. Focal spots also exist in the hindwing, although they are not seen without removal of the forewing. 

### 2.2. Mechanical Load Tests

Pupae were subjected to mechanical load tests using a Lutron Electronics Enterprise Fruit Hardness Tester FR-5105 (Taipei, Taiwan). A pupa was placed on the plain surface of the laboratory bench ventral side up. A gauge probe was placed vertically at the specific position on the surface of the pupa and was slowly pushed down manually until the treated pupa ruptured (Figure 1D,E). The peak hold mode was used to obtain the maximal force at rupture, which was considered the hardness of pupae. When a pupa did not rupture due to a soft cuticle (in the case of cold shock treatment), the maximal value recorded during the vertical probe movement was considered the maximal force and hardness of the pupae.

### 2.3. Chemical Injections

Chemical injections were performed at the abdomen within 6 h after pupation using an Ito Microsyringe MS-05 (Fuji, Shizuoka, Japan). The following chemicals were used for injections into the pupae: fluorescent brightener 28 (FB28) (supplied as 25% aqueous solution) (Sigma-Aldrich, St. Louis, MO, USA), sodium tungstate (Sigma-Aldrich), heparin sodium (FUJIFILM Wako Pure Chemical, Osaka, Japan), dextran sulfate sodium salt from *Leuconostoc* spp. (Sigma-Aldrich), chlorfluazuron (FUJIFILM Wako), captan (FUJIFILM Wako), Congo red (MP Biomedicals, Solon, OH, USA), polyoxyin B (AOBIOUS, Gloucester, MA, USA), polyoxyin D from *Streptomyces cacaoi* var. *asoensis* (KAKEN PHARMACEUTICAL, Tokyo, Japan) and sodium chloride (Wako). Chlorfluazuron and captan were first dissolved in ethyl acetate and further diluted in deionized water. Other chemicals were directly dissolved or diluted in deionized water.

FB28 has many synonyms, such as Fluostain I, Calcofluor White LRP, ST, or M2R, and Tinopal LPW or UNPA-GX. FB28 binds to polysaccharides, including chitin [43,44]. In Lepidoptera, FB28 has been tested for the enhancement of viral infection in agricultural pest moths due to inhibition of the cuticular peritrophic matrix membrane [45,46,47,48]. It has been used recently for chitin detection in the fruit fly [49]. Chlorfluazuron, captan, and Congo red have also been used similarly [48,50,51]. Polyoxyin B and polyoxyin D have been used similarly as inhibitors of chitin synthesis in insects [52,53,54]. They are known to cause ecdysis failure in larvae [54].

### 2.4. Covering Material Experiments

For the forewing lift operation, first reported in 2009 [55] and used in subsequent studies, the right forewing was carefully lifted with forceps immediately after pupation as in previous studies [55,56]. For covering material experiments, the ventral forewing and the dorsal hindwing surfaces exposed by the operation were covered with a piece of transparent plastic film (polyvinylidene chloride; Kurewrap, Kureha, Tokyo, Japan), a glass plate (frosted glass slide; AS ONE, Osaka, Japan), or silicone glassine paper (CGC Japan, Tokyo, Japan), according to previous studies [40,41]. These materials are collectively called covering materials or contact materials. Plastic film and glass plate are relatively hydrophilic, whereas silicone glassine paper is relatively hydrophobic based on water contact angles [41].

### 2.5. Developmental Imaging

For observations of developing wings, the right forewing was lifted as above and covered with a piece of plastic film, after which FB28 (7.5% in deionized water) was injected. Pictures of specimens were then taken using an Olympus digital camera Tough TG-6 (Tokyo, Japan). FB28 fluorescent signals were imaged under ultraviolet light at 365 nm from a NICHIA UV-LED (Anan, Tokushima, Japan). The maximal excitation and emission wavelengths of FB28 are 365 nm and 435 nm, respectively.

For real-time confocal in vivo imaging, after the forewing-lift operation, the wing surfaces were tentatively covered with a piece of plastic film, and FB28 (7.5%, 1.0 μL) was injected. Plastic film was then removed, and MitoRed (DOJINDO LABORATORIES, Mashiki, Kumamoto, Japan) and BODIPY FL C_5_-ceramide complexed to BSA (Molecular Probes, Eugene, OR, USA) were loaded by the sandwich method [57]. After the loading process, the wing surfaces were covered with a piece of plastic film again. One day after the treatments, the plastic film was removed, the wing surfaces were placed on a glass plate, and confocal images were obtained.

Confocal images were obtained using a Nikon A1^+^ confocal microscope (Tokyo, Japan) according to previous studies [57]. For the detection of MitoRed staining, an excitation laser at 561 nm and an emission filter at 595/25 nm were used. For the detection of BODIPY FL C_5_-ceramide staining, an excitation laser at 488 nm and emission filter at 520/25 nm were used. For detection of FB28 staining, an excitation laser at 405 nm and an emission filter at 450/25 nm were used. This confocal system had a high-resolution galvano scanner and was operated by Nikon NIS Elements software. Images were edited by this software to obtain three-dimensional reconstruction images.

### 2.6. Statistical Analysis

To compare hardness values obtained from mechanical load tests, Student’s *t*-test (unpaired, two-tailed) was performed using Microsoft Excel (Office 365) and JSTAT 13.0 (Yokohama, Japan). For simplicity, we reported *p*-values that were not corrected with Bonferroni or other methods.

## 3. Results

### 3.1. Mechanical Load Test for Cuticle Hardness

On the basis of previous results on TS-type modifications, we hypothesized that chemical modification inducers such as tungstate and heparin act as inhibitors of cuticle formation, which then affects color pattern modifications in adult butterfly wings. To test this pupal cuticle hypothesis, we first performed a mechanical load test of pupae after modification-inducing treatments to infer the potential influence of the modification inducers on cuticular exoskeleton formation or sclerotization.

**Figure 2:**
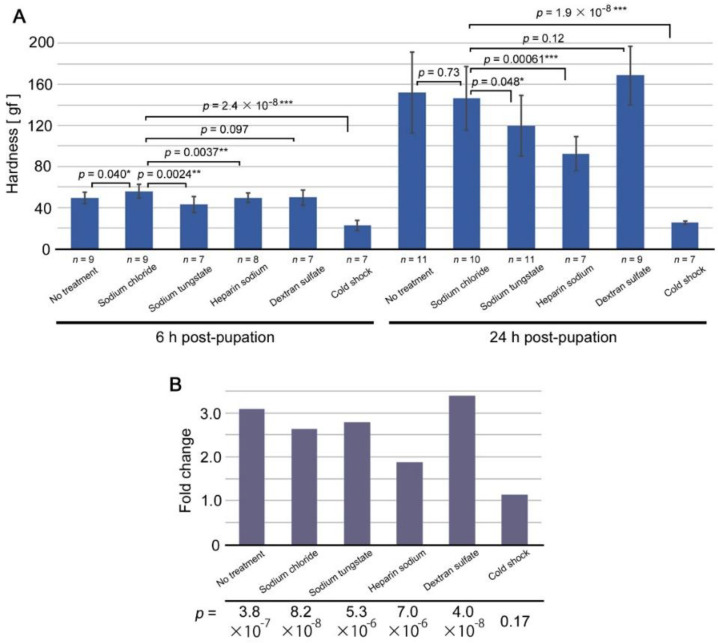
Results of mechanical load tests for pupae treated with various modification-inducing treatments. (**A**) Pupal hardness at 6 h and 24 h post-pupation under modification-inducing treatments. The hardness is expressed in gram-force. The number of treated pupae is shown for each treatment. Sodium chloride treatment was compared with other treatment modes (including no treatment). Asterisks indicate statistical significance: * *p* < 0.05; ** *p* < 0.01; *** *p* < 0.001. (**B**) Fold change values from 6 h to 24 h post-pupation. At the bottom, *p*-values between 6-h and 12-h hardness values are shown.

In pupae at 6 h post-pupation (Figure 2A, left), injection of sodium chloride, which does not induce any modification [30], slightly increased pupal hardness in comparison to no treatment. We thus used pupae injected with sodium chloride as a control group for comparison. Pupae injected with sodium tungstate or heparin sodium showed a significant decrease in hardness. Pupae injected with dextran sulfate, a reversed TS-type modification inducer, did not show a significant difference. Notably, pupae treated with cold shock were very soft, showing a highly significant difference in hardness.

In pupae at 24 h post-pupation (Figure 2A, right), pupae injected with sodium chloride did not show a significant difference in hardness from those with no treatment, but we used the former as a control group for comparison. Pupae injected with sodium tungstate showed a significant decrease in hardness but with a *p*-value near the border of *p* = 0.05. Pupae injected with heparin sodium showed a significant decrease with a much lower *p*-value. Pupae injected with dextran sulfate showed an increase, but it was not statistically significant. Cold shock treatment made pupae much softer than those injected with sodium chloride to a more significant degree. It appeared that low temperature and other treatments inhibited a process of cuticle formation, possibly sclerotization, with the exception of dextran sulfate.

Comparisons between 6 h and 24 h hardness values showed that the differences were highly significant except in cold shock (Figure 2B), indicating that the pupal exoskeleton became rigid during this period of time in most treatments. Cold shock appeared to severely inhibit the sclerotization process, but other treatments appeared to be less severe. This is probably because cold shock inhibited sclerotization throughout the body, whereas other treatments inhibited sclerotization at specific sites inside the body. Fold change values of hardness from 6 h to 24 h post-pupation indicated that heparin sodium treatment was lower than sodium chloride treatment. Sodium tungstate was slightly higher than sodium chloride and slightly lower than no treatment. Dextran sulfate was the highest. These results were consistent with the hypothesis that modification-inducing treatments work by inhibiting cuticle formation or sclerotization.

### 3.2. Inhibitors of Cuticle Formation Tested: Discovery of FB28 as a Modification Inducer

**Figure 3:**
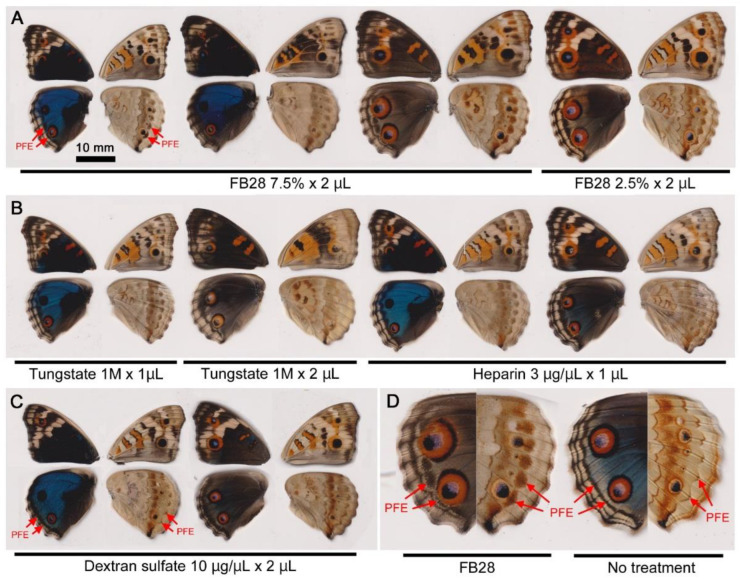
Color pattern modifications induced by FB28 and other modification inducers. (**A**) FB28. (**B**) Previously known modification inducers, tungstate and heparin. These treatments cause similar modifications of PFEs toward the proximal positions (closer to eyespots). (**C**) Dextran sulfate. This treatment acts on the thickening and dislocation of PFEs (farther from eyespots). (**D**) Comparison between a hindwing treated with FB28 and a hindwing without treatment. The dorsal (**left**) and ventral (**right**) sides of the hindwing are shown. PFEs are indicated by arrows.

There are known inhibitors of cuticle formation in insects. We reasoned that there may be other chemicals that induce TS-type modifications if the pupal cuticle hypothesis is correct. Here, in addition to known modification inducers (i.e., tungstate, heparin, and dextran sulfate), we tested several chemicals known to inhibit cuticle formation in insects (Table 1). Among them, only fluorescent brightener 28 (FB28) induced modifications. The modification rate (MR) appeared to be dose-dependent (Table 1). The modified color patterns were similar, if not identical, to those of sodium tungstate and heparin (Figure 3). Most notably, PFEs were thickened and dislocated toward eyespots (Figure 3).

**Figure 4:**
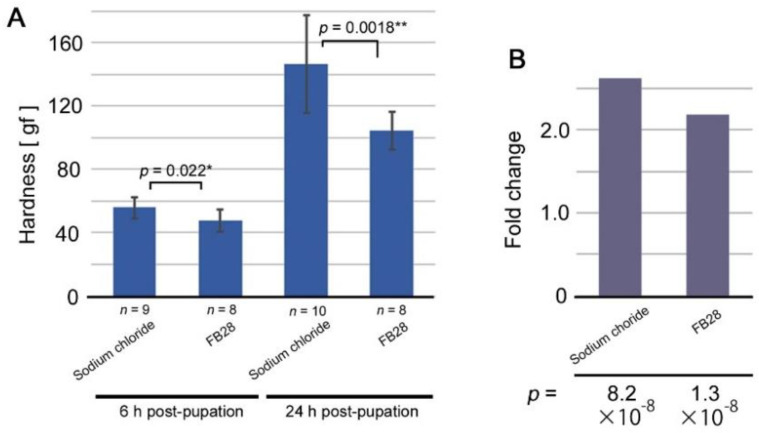
Results of mechanical load tests for pupae treated with FB28. (**A**) Pupal hardness 6 h and 24 h post-pupation. The hardness is expressed in gram-force. The number of treated pupae is shown for each treatment. Sodium chloride treatment was compared with FB28 treatment. Asterisks indicate statistical significance: * *p* < 0.05; ** *p* < 0.01. (**B**) Fold change values from 6 h to 24 h post-pupation. At the bottom, *p*-values between 6-h and 12-h hardness values are shown.

We then performed mechanical load tests for pupae treated with FB28. The results were similar to those of tungstate (Figure 4). In comparison to sodium chloride, FB28-treated pupae became significantly softer both at 6 h post-pupation and at 24 h post-pupation (Figure 4A). The fold-change value of FB28 from 6 h to 24 h post-pupation was also lower than that of sodium chloride, although both showed a significant increase from 6 h to 24 h post-pupation (Figure 4B). These results suggest that FB28 likely induced modifications through the same molecular mechanisms as sodium tungstate and heparin sodium.

### 3.3. Sites of FB28 Staining

**Figure 5:**
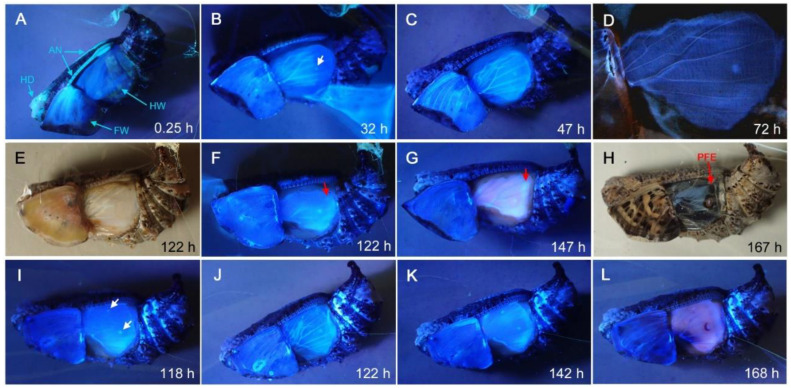
FB28 fluorescent signals under ultraviolet light from forewing-lifted pupae after injection. Post-injection hours are indicated. (**A**–**D**) A pupa. HD, AN, FW, and HW indicate the head, antennae, forewing and hindwing, respectively. The white arrow in B indicates a pupal cuticle focal spot. (**E**–**H**) Another pupa. E and H are under white light. The red arrows in F and G indicate fluorescent signals at the peripheral area, which may correspond to PFE in H. (**I**–**L**) Yet another pupa. Arrows in I indicate pupal cuticle focal spots, which correspond to the foci of eyespots shown in (**K**,**L**).

Because FB28 is a fluorescent chemical, we observed where FB28 accumulated in the body of pupae. Under the forewing-lift configuration, fluorescent signals were detected in the head, antennae, and wings immediately after injection (Figure 5A). One day post-injection, fluorescent signals were detected in wing veins and at the pupal cuticle focal spot in the hindwing, corresponding to the cuticle just above the prospective eyespot focal organizing cells (Figure 5B). The fluorescent signal at the pupal cuticle focal spots appeared to become more intense two days and three days post-injection (Figure 5C,D). Five days post-injection, peripheral areas of the hindwing also became fluorescent (Figure 5E–L).

**Figure 6:**
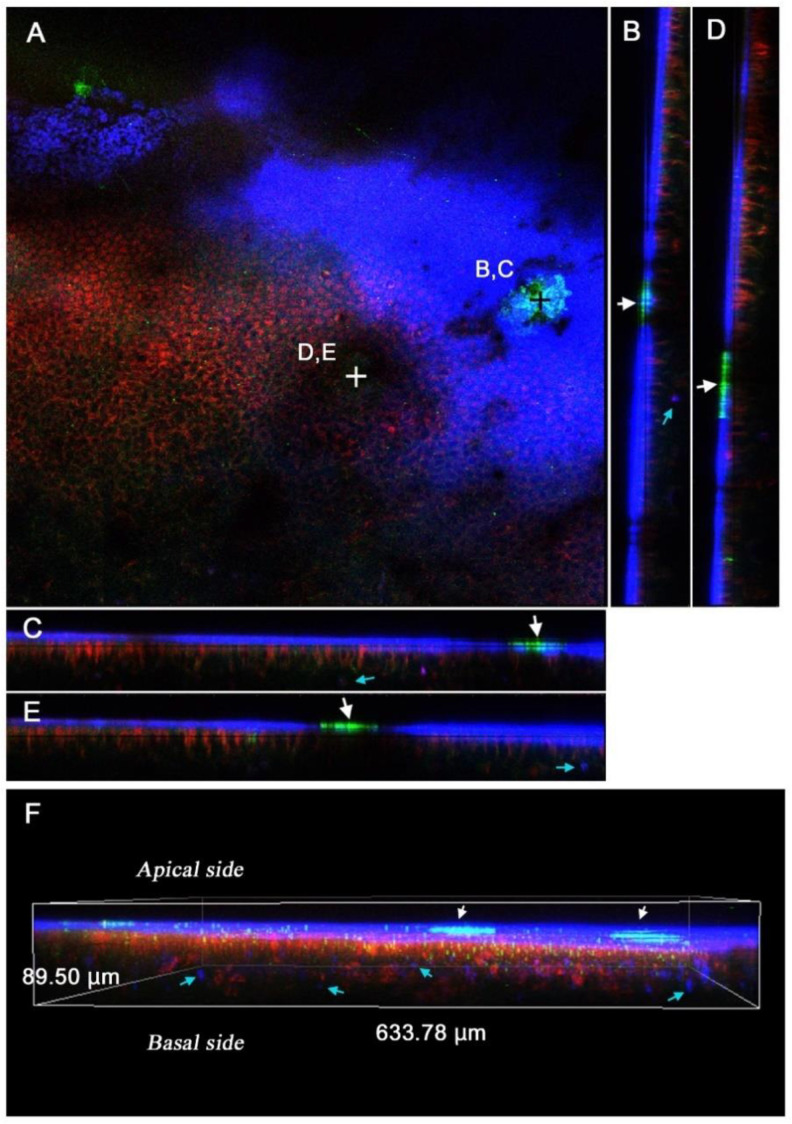
Confocal optical sections and three-dimensional reconstructions of the pupal hindwings stained with MitoRed for mitochondria (red) and BODIPY FL C_5_-ceramide for membranous structures (green) together with FB28 (blue). White arrows indicate the pupal cuticle focal spot. Blue arrows indicate FB28-positive cells below or between the epithelial cells. (**A**) An optical horizontal section at the surface area of the hindwing. The light blue-green area on the right is a pupal cuticle focal spot embedded within the procuticle layer. A black cross at the center of the spot indicates positions of cross-sectioning lines for B and C. Red signals on the left are mitochondria below the cuticle due to a tilt of the sample. A white cross at the center of this panel indicates the positions of the cross-sectioning lines for D and E. At the position of the white cross, another spot is located (out of focus). (**B**,**C**) Cross sections of A that transverse the spot. (**D,E**) Cross sections of A that transverse another spot (not visible in A). (**F**) A three-dimensional reconstruction image of A.

To further verify the sites of FB28 fluorescence, real-time confocal in vivo images were obtained under the forewing-lift configuration. We stained epithelial cells with MitoRed (for mitochondria) and BODIPY FL C_5_-ceramide (for membranous structures). It has been reported that mitochondria are located at the surface of wing epithelial cells [57]. FB28 signals were mainly located in the procuticle (endocuticle) as a thin blue layer, just above the mitochondrial (red) and membrane (green) signals (*n* = 3) (Figure 6). Epithelial cells per se were not stained with FB28. FB28-positive cells were sparingly detected at the deeper level, but they are probably hemocytes [57]. Notably, the pupal cuticle focal spots were stained intensively with FB28. The pupal cuticle focal spots also emitted green fluorescence. This signal is probably autofluorescence [42].

### 3.4. FB28 on the Pupal and Adult Cuticle Structures

**Figure 7:**
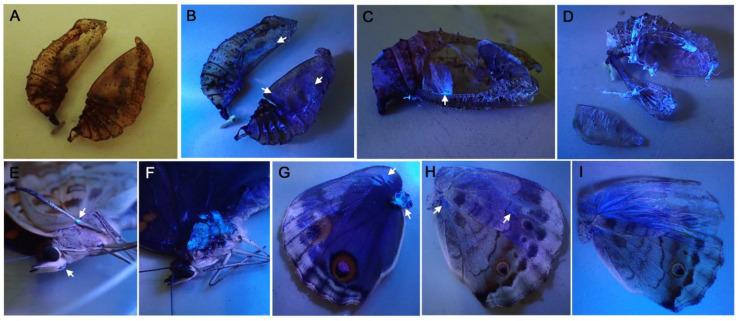
FB28 fluorescent signals from the post-pupation pupal and adult cuticle structures. (**A**) A pupal case of sagittal cut under white light. (**B**) The same specimen of A under ultraviolet light. Blue fluorescent signals are detected from many parts, but arrows indicate representative ones. (**C**). A pupal case with the forewing-lift operation. An arrow indicates the FB28 fluorescent signal from the thin cuticle covering the dorsal surface of the hindwing. (**D**) A dissected pupal case. The inner side of the cuticle covering the dorsal forewing is shown at the bottom left side of this panel, showing blue fluorescence. (**E**) Leg joint showing blue fluorescence. (**F**) Thorax muscle after removal of the exoskeleton, showing strong blue fluorescence. (**G**) Dorsal side of an adult hindwing. Cover scales at the anterior part were removed. Arrows indicate wing joint and wing base showing blue signals. (**H**) Ventral side of an adult hindwing. Arrows indicate fluorescent signals from the wing basal membrane due to scale removal. (**I**) Ventral side of an adult hindwing. Fluorescent signals were detected from the wing basal membrane. Cover and ground scales at the anterior part were removed.

After eclosion, we examined the pupal cuticle (post-eclosion pupal case) for FB28 fluorescence. FB28 fluorescence was readily detected from various inner surfaces of the pupal case (Figure 7A–D). Thin cuticles covering the dorsal hindwing were fluorescent (Figure 7C), confirming the previous pupal observations. Interestingly, adult cuticles were also stained with FB28; a part of the leg (Figure 7E), thorax muscle (Figure 7F), and wing basal membrane (Figure 7G). These results suggest that FB28 binds to pupal and adult procuticles throughout the body when injected.

### 3.5. Covering Materials and FB28

**Figure 8:**
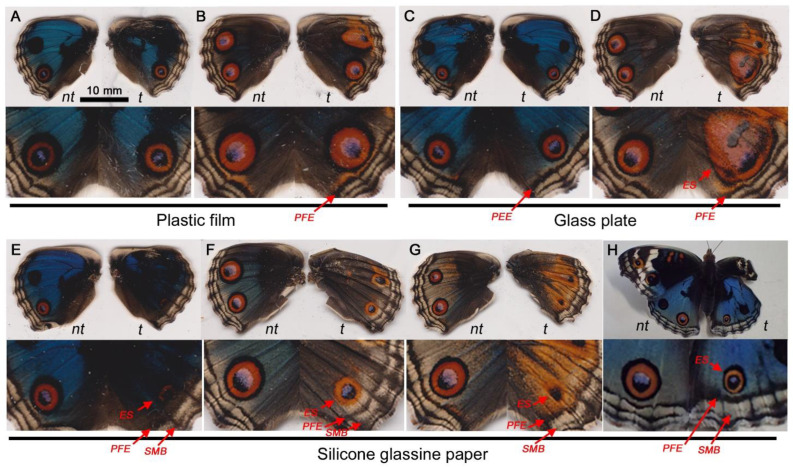
Color pattern modifications induced by covering materials. Only the right dorsal hindwing was treated with a covering material (*t*), and the left hindwing was not treated (*nt*). The top panels show the entire dorsal hindwings, and the bottom panels show magnification of the posterior eyespots on the dorsal hindwings. Eyespot (ES), parafocal element (PFE), and submarginal band (SMB) are indicated by red arrows. (**A**,**B**) Individuals treated with plastic film. (**C**,**D**) Individuals treated with glass plate. (**E**–**H**) Individuals treated with silicone glassine paper.

Here, we examined the effects of FB28 when an artificial covering material was placed over wing epithelial cells, another type of modification-inducing treatment. The effects of covering materials on eyespot formation have been reported [40,41], but to confirm these findings, here, we first replicated the contact-induced modifications without FB28 (Figure 8). As expected, plastic film, a relatively hydrophilic material, did not increase or decrease the eyespot size in visual inspections (*n* = 6) (Figure 8A,B). Glass plate, another relatively hydrophilic material, induced either no change (*n* = 3) or enlargement (*n* = 1) of the eyespot in size (*n* = 4) (Figure 8C,D). Silicone glassine paper, a relatively hydrophobic material, greatly decreased the eyespot size (*n* = 4) (Figure 8E–H). Simultaneously, PFEs were thickened and dislocated toward eyespots, a unique feature of TS-type modifications (Figure 8E–H). These effects have been interpreted as evidence for the functional importance of the direct contact of epithelial cells with a solid cuticle (or an artificial covering material) for morphogenic signal propagation [40,41]. However, the “direct” contact may still have room for ECM molecules between the solid cuticle (or a covering material) and the wing epithelial cells.

**Figure 9:**
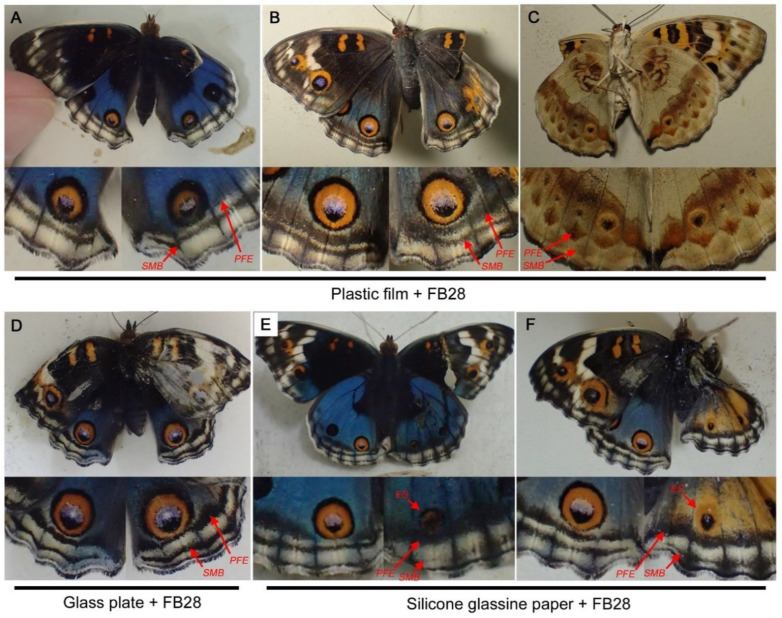
Double treatments with a covering material and the injection of FB28. Only the right dorsal hindwing was treated with a covering material. The top panels show the dorsal view of the treated individuals (the ventral view in C), and the bottom panels show magnification of the posterior eyespots on the hindwings. Eyespot (ES), parafocal element (PFE), and submarginal band (SMB) are indicated by red arrows. (**A**–**C**) Individuals treated with plastic film and FB28. (**D**) Individuals treated with glass plate and FB28. (**E,F**) Individuals treated with silicone glassine paper and FB28.

Taking the results of the covering materials above into account, pupae were simultaneously treated with a covering material and with an injection of FB28 (Figure 9). For these double treatments, plastic film (*n* = 7) (Figure 9A–C), glass plate (*n* = 3) (Figure 9D), or silicone glassine paper (*n* = 11) (Figure 9E, F) were used, as shown in Figure 8. In the plastic film treatment with FB28, eyespots showed either no change (*n* = 5) or enlargement (*n* = 2). In the glass treatment with FB28, the eyespot showed no change (*n* = 3). Nevertheless, in these individuals, thickening and dislocation of PFEs were observed both in the treated right hindwing and in the non-treated left hindwing (Figure 9A–D). Because plastic film and glass plate in most cases did not affect eyespots and PFEs, similar to natural cuticle coverage, it was certain that FB28 acted on both treated and non-treated wings. When silicone glassine paper was used, all individuals (*n* = 11) showed eyespot size reduction in the treated right hindwing (Figure 9E,F), which was not interrupted by FB28. In this case, it was not certain that FB28 acted on the PFEs in the treated right hindwing because both PFEs and eyespots can be modified by silicone glassine paper.

We also performed double treatment experiments using covering materials and sodium tungstate. Plastic film showed no change in eyespot size (*n* = 3), glass plate showed either no change (*n* = 2) or enlargement (*n* = 1), and silicone glassine paper showed reduction (*n* = 2). In all of these modified individuals, TS-type modifications were induced both in the treated right hindwing and in the non-treated left hindwing (not shown). The results of FB28 and tungstate were indistinguishable.

### 3.6. Covering Materials and Dextran Sulfate

**Figure 10:**
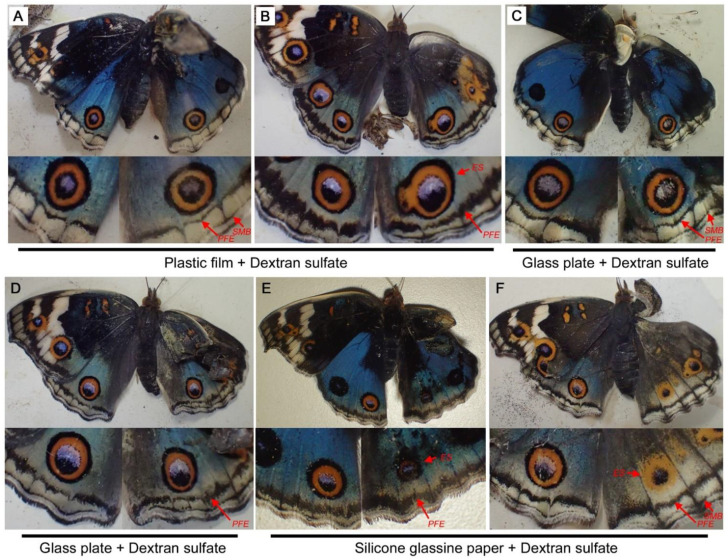
Double treatments with a covering material and dextran sulfate. Only the right dorsal hindwing was treated with a covering material. The top panels show the dorsal view of treated individuals, and the bottom panels show magnification of the posterior eyespots on the dorsal hindwings. Eyespot (ES), parafocal element (PFE), and submarginal band (SMB) are indicated by red arrows. (**A**,**B**) Individuals treated with plastic film and dextran sulfate. (**C**,**D**) Individuals treated with glass plate and dextran sulfate. (**E**,**F**) Individuals treated with silicone glassine paper and dextran sulfate.

Among modification inducers, dextran sulfate is unique in that its induced modifications are different from those of the typical TS type; PFEs are selectively thinned or enhanced [34] (see Figure 3C). Here, dextran sulfate was applied together with a covering material. When plastic film was used (*n* = 6), the eyespot size was not changed (*n* = 4) or enlarged (*n* = 2) (Figure 10A,B). When glass plate was used (*n* = 5), none of the individuals showed an eyespot size change (Figure 10C,D). Regardless of eyespot changes, comparison between the treated right and non-treated left hindwings indicated that the effects of dextran sulfate on the PFEs appeared to be slightly inhibited by these covering materials. Nonetheless, dextran sulfate worked on the right hindwings with these covering materials.

In the case of silicone glassine paper (*n* = 8), all individuals showed considerably reduced eyespots (Figure 10E,F). As expected from the results of the singular dextran sulfate treatment, PFEs responded to dextran sulfate. Unexpectedly, however, PFEs did not respond to silicone glassine paper when FB28 was injected. Comparison between the right and left hindwings indicated that the effects of dextran sulfate on the PFEs appeared to be slightly inhibited by silicone glassine paper, as in the cases of the plastic film and glass slide.

## 4. Discussion

### 4.1. The Pupal Cuticle Hypothesis

The present study hypothesized the importance of the apical pupal cuticle and/or ECM for wing color pattern formation in butterflies. This “pupal cuticle hypothesis” is not new, as the importance of the pupal cuticle in color pattern determination has been repeatedly implicated [6,40,41,42,58]. Crucial supporting data for this hypothesis have been presented by forewing-lift experiments with various covering materials [40,41], some of which were reproduced in the present study. More importantly, we presented additional evidence here for the pupal cuticle hypothesis in terms of the correlational relationship of modification-inducing treatments with the mechanical hardness of pupae, an indication of cuticle formation or sclerotization. We discovered that most modification inducers, except dextran sulfate, made the pupal cuticular exoskeleton less rigid. These results may be because the treatments delayed sclerotization, considering the significant differences in hardness between 6 h and 24 h post-pupation. By these treatments, ECM molecules may also be affected because their functions may be dependent on cuticle status. 

Among the treatments tested, cold shock was by far the most effective in making the pupal cuticle less rigid, but its effectiveness in modification induction was not more than other chemical inducers, suggesting that chemical inducers are more specific to modification induction than cold shock. It is interesting to note that non-specific cold shock treatment results in a decrease in hardness and in specific changes in wing color patterns. In other words, non-specificity (i.e., cold shock) induces specificity (i.e., modifications), and these two factors are bridged by cuticle hardness. 

In contrast, dextran sulfate made the pupal cuticle slightly less rigid at 6 h post-pupation but made it more rigid at 24 h post-pupation, although these results were not statistically significant. Dextran sulfate might have first decelerated and then accelerated sclerotization, although its mechanism is unclear. This possible acceleration is consistent with the previous interpretation that dextran sulfate is an accelerator of morphogenic signal propagation [35], although the original study that discovered dextran sulfate as a modification inducer has interpreted their data in accordance with the classical gradient model [34]. In any case, the fact that dextran sulfate made the pupal cuticle more rigid at 24 h post-pupation in contrast to other modification inducers supports the idea that this mechanical load test is not an indication of general stress response but an indication of the modification-inducing activity of these treatments.

More importantly, what is new in the present study is that FB28 was discovered as a new modification inducer based on the pupal cuticle hypothesis. This discovery itself suggests that the hypothesis is not on the wrong track. The FB28-induced modifications were very similar to those induced by cold shock, tungstate, and heparin in *J. orithya*. Furthermore, FB28 made pupae less rigid, as did other modification inducers. Fortunately, FB28 is a fluorescent substance. Taking advantage of this fluorescent nature of FB28, we localized the sites where FB28 accumulated in wings. The FB28-positive sites are likely the target sites of modification inducers. Although the present study focused on the dorsal hindwing, all four wing surfaces are likely covered with a cuticle and its associated ECM, and we believe that all four wing surfaces are color-patterned essentially in the same way.

However, it is difficult to distinguish the two sites, the cuticle itself or its facing ECM (see below), which are responsible for the color pattern modifications, because chitin is an ECM molecule and also a component of the cuticle [59] and because this ECM space may be very small before apolysis. Cuticle hardness and covering materials may influence the composition of the ECM, resulting in the modifications. Alternatively, cuticle hardness may merely be an indication of ECM functionality. This line of argument is consistent with the notion that molecular morphogens such as Wnt travel in the apical ECM with the help of polysaccharides [60]. 

It may be important to stress here that the contact-induced modifications with covering materials completely differ from damage-induced color pattern changes. The former does not require any physical damage to epithelial cells and is dependent on the hydrophobicity of the covering materials [40,41]. The latter requires deep damage to induce site-specific changes [1,6]. The induced modification patterns are also very different in these two treatments. The former induces changes in an area covered by a covering material and is similar to the modifications induced by tungstate, heparin, and cold shock in terms of the border symmetry system (i.e., eyespots and PFEs) [40,41]. The latter induces a site-specific spot when a background area is damaged or reduces an eyespot in size when the center of the prospective eyespot is damaged [1,6].

### 4.2. Chitin: The Potential Site of Action of Various Modification Inducers

We observed that FB28 reached the wings a few minutes after injection and that there was an FB28-positive layer, likely a chitin-containing procuticle (endocuticle), immediately above the apical plasma membrane of the wing epithelial cells. FB28-positive fluorescence was scarcely observed in the epithelial cell layer. Rare FB28-positive cells are likely hemocytes and not epithelial cells. Thus, the most likely interpretation is that FB28 diffuses in the apical ECM and modifies morphogenic signals there.

It should be noted that the images of the dorsal hindwing were obtained from pupae, the wing surface of which had been covered with a piece of plastic film for one day before imaging. It is understood that cuticle secretion and formation may be kept at a low level on the wing surface when a covering material is placed, allowing for optical in vivo observations [40,41]. However, based on the confocal images (Figure 6) and the post-eclosion pupal cuticle (Figure 7C), it is clear that plastic film (and other covering materials) could not completely inhibit cuticle formation. It is likely that the covering materials did not make direct contact with the apical plasma membrane of the wing epithelial cells. Instead, covering materials probably affected the procuticle. Moreover, double treatments with a covering material and a chemical injection revealed that these treatments acted simultaneously, suggesting the functional existence of a physical extracellular space between the pupal cuticle and the wing epithelium.

In fact, there is likely to be an ECM, which may be called the “adhesion zone” [61], above the apical surface of the wing epithelial cells and below the procuticle layer per se, and this adhesion zone is accessible from the basal side via hemolymph. There may be a direct route via loose horizontal cellular interactions at the early pupal stage [57]. Alternatively, FB28 may be absorbed by the epithelial cells at the basal side and secreted into the apical extracellular side. However, the latter is unlikely because we did not observe FB28-positive epithelial cells.

The interpretation above is consistent with the fact that FB28 binds to chitin [43,44], an important constituent polysaccharide of the procuticle in insects [62]. Chitin is required for cuticular sclerotization [61], which explains the present results of the mechanical load test. It follows that not only FB28 but also other various modification-inducing treatments may inhibit cuticle formation by changing chitin biochemistry on the apical surface of the wing epithelial cells. Tungstate, when combined with hydrogen peroxide, is known to catalyze the oxidation and degradation of polysaccharides [63,64,65]. Heparin and other polysaccharides that function as modification inducers [34] may act as competitive inhibitors of chitin. Cold shock may non-specifically inhibit chitin biochemistry for sclerotization but may upregulate chitinases for chitin degradation [66]. Cold shock may also induce a humoral factor [37], which may act on chitin prematurely. Relatively hydrophilic covering materials, such as plastic film and glass plate, may provide an appropriate surface for chitin binding and stabilization required for morphogenic signals from organizers to propagate normally [40,41]. Relatively hydrophobic covering materials such as silicone glassine paper may not be able to serve as a binding substrate for chitin. Therefore, most modification inducers of various types of chemicals appear to consistently act on chitin. An acid carboxypeptidase [33] is difficult to understand from the viewpoint of chitin at present, but this enzyme may degrade chitin-associated enzymes or structural proteins in the adhesion zone.

FB28 was concentrated at the pupal cuticle focal spots above the organizing focal cells for eyespots. These spots appear to have different compositions from other procuticle areas because the spots were stained differently from the rest and because the spots emitted green autofluorescence in addition to blue fluorescence from FB28. These green autofluorescent signals from the pupal cuticle focal spot have already been reported [42]. Cells at the organizing centers may have the ability to construct pupal cuticle focal spots with unique compositions, which may be important for morphogenic signal propagation. This line of argument states that the pupal cuticle focal spots are not a mere non-functional structure just for camouflage but a developmentally important functional apparatus. This notion is consistent with the fact that the spots are widely conserved in various species of Nymphalidae and in other families of butterflies [42]. In addition, wing veins were FB28-positive. It should be noted that wing veins may function as organizers for vein-dependent color patterns [1]. 

### 4.3. Chitin and Morphogenic Signals

How does chitin contribute to morphogenic signal propagation? During the period of color pattern determination, chitin production from epithelial cells may continue, and thus cuticular and ECM environments may change continuously. How such dynamic chitin in the ECM assists morphogen molecules to propagate is enigmatic. Heparin-induced modifications are considered a *Wnt* gain-of-function phenotype [13,19,67,68], but modification inducers including heparin are generally considered to repress propagation of morphogenic signals based on color pattern analysis [35]. Moreover, heparin and other modification inducers cause wing-wide changes regardless of *WntA* expression that is specific to limited elements. These observations can be explained only when Wnt is a negative regulator of unknown morphogenic signals. This interpretation is consistent with the fact that *WntA* loss-of-function mutants [13,19] show high similarity to individuals treated with dextran sulfate, an accelerator of morphogenic signal propagation [35].

Based on a chitin synthase mutant, chitin is required for cells to attach to the cuticle [61]. In insects, adult morphology develops from pupal tissues attached to the pupal cuticle before apolysis. During adult development, the pupal cuticle may function as a template. This epithelial cell attachment to chitin may be required for propagation of morphogenic signals for color pattern determination. The binding of the apical surface of the epithelium with an opposing cuticle surface via chitin may provide mechanical support for signal propagation. Modification inducers may act especially at the pupal cuticle focal spots or their facing ECM to inhibit morphogenic signal release and propagation. It should be noted that cellular attachment to the cuticle via chitin and the ECM in the adhesion zone may coexist because of three-dimensional cuticular and epithelial surfaces at the molecular level. 

### 4.4. Behaviors of Eyespots and Parafocal Elements

Different behaviors of eyespots and PFEs in the double treatments with silicone glassine paper and dextran sulfate need to be discussed. Both eyespot and PFE signals cannot proceed under silicone glassine paper [40,41], as also shown in the present study. Functional chitin (stabilized and ready for binding to cells) may be depleted by the treatment with silicone glassine paper, although its mechanism is unclear. However, PFE signals (but not eyespot signals) can proceed even under silicone glassine paper treatment when dextran sulfate is present. This result may be expected, considering that dextran sulfate has been considered an accelerator of morphogenic signal propagation [35]. A high concentration of dextran sulfate in the ECM may accelerate the propagation of morphogenic signals for PFEs as an augmentation of chitin function. Once propagated, the PFE signals may be able to propagate further with the help of dextran sulfate even when functional chitin is not available.

### 4.5. Chitin-Related Molecules and Transcription Factors

This study highlights the importance of chitin in the extracellular milieu in insect morphogenesis. The insect cuticle is composed of chitin and structural proteins and is known to play critical roles in insect morphogenesis [61,62,63,64]. Many chitin synthases, chitinases, and other cuticle-associated molecules are known to function in insect cuticle formation [61,69,70,71,72,73,74]. A transcriptome study of eyespots has reported that “chitinase-like” and “cuticle 3-like” genes, among others, are significantly upregulated at prospective eyespots [17]. Mosaic knockouts of *laccase2*, which encodes an important enzyme for cuticle formation, have been reported [27]. Although their phenotypes are not spectacular, this gene is likely involved in color pattern formation in adult wings [27].

Additionally, important transcription factors are known to be expressed just below the pupal cuticle marks (markings) [26]. These transcription factors are expressed even in wing compartments where eyespots are non-existent in adult wings [26]. This means that the expression of these transcription factors does not necessarily result in eyespot formation. Eyespot existence and non-existence may be related to differences in the size of the pupal cuticle focal spots [58], which can be attributed to chitin synthase and other cuticle-related enzymes. The existence or non-existence of eyespots may also be determined by the heterochronic relationship with adjacent eyespots [41]. Organizing cells at the center of the prospective eyespot are relatively large and probably undergo several nuclear divisions [55], which may produce pupal cuticle focal spots and other cuticular structures.

### 4.6. Molecular (Chemical) Morphogens and Mechanical (Physical) Morphogens

Molecular morphogens such as Wnt proteins are certainly important, but a simple gradient model for positional information based on morphogenic diffusion may not work well in the butterfly color pattern system [75,76,77,78]. To mention an example, the discovery of overpainting of scale colors indicated a dynamic interaction between the dark-inducing and light-inducing signals [56,77]. To circumvent this problem, one possibility is that molecular morphogens may be delivered through intercellular connections [79,80,81,82,83] in butterflies because such structures have been observed in developing pupal wing tissues [57]. In an in vivo imaging study [57], the live cellular structures of the wing epithelium were revealed, which may be considered a pseudostratified columnar epithelium similar to olfactory epithelia [84], and fine cellular connections have been reported as epidermal feet (see Figure 7e in Ohno and Otaki (2015) [57]). Interestingly, mitochondrial signals have been detected within the structures [57], suggesting that they may be tunneling nanotubes [82,83]. At deeper levels, horizontal cellular connections have also been reported as cytonemes (see Figures 8 and 10 in Ohno and Otaki (2015) [57]). From the viewpoint of molecular morphogens, the importance of chitin in color pattern determination may simply be interpreted as a requirement of cytonemes or other connecting structures for scaffolds. An important morphogen, Wnt, is known to travel in the apical side of the wing epithelium in *Drosophila* [60]. If no cellular connecting structures are employed in Wnt propagation, Wnt may travel by binding to ECM polysaccharides such as chitin, which may partly overcome the problems associated with a diffusion model.

In addition to conventional molecular (chemical) morphogens, there is a possibility that mechanical distortion of the epithelium serves as a non-molecular (physical) morphogen to induce subsequent molecular changes, in accordance with the distortion hypothesis for butterfly wing color pattern formation [6]. The distortion hypothesis has been proposed to circumvent several problems of chemical morphogens and to explain unexplained morphological and physiological features associated with color pattern development [6]. Here, mechanical (physical) morphogen is defined as a distortion force in the tissue, generated by cellular dynamics of organizing cells, that can induce differentiation in surrounding immature cells. The supposed physical morphogen is compatible with the conventional molecular morphogen. Mechanical signals may be assisted by ECM molecules such as polysaccharides or proteins and would ultimately be converted to molecular signals through force sensing receptors. The morphogen receptor cells may be located away from the organizing cells.

Hypothetical distortion signals are considered as important in achieving long-range interactive signaling in butterfly color pattern determination. Long-range and interactive signals have been implicated by covering material experiments for inducing eyespots in eyespot-less compartments [41]. Accordingly, an integrative model has been proposed as the induction model for positional information [6,75,76,77,78]. In this model, chitin in the pupal cuticle functions as a cellular scaffold. The pupal cuticle focal spots, a specialized cuticle structure, may ensure that organizing cells bind tightly to generate horizontal force through volumetric expansion of cells.

## 5. Conclusions

Based on the pupal cuticle hypothesis, we found that known modification inducers changed the hardness of pupae, and we discovered FB28 as a new modification inducer. FB28 made the pupal exoskeleton less rigid, as did other modification inducers. Because the FB28-stained procuticle (endocuticle) layer (including the pupal cuticle focal spot) is immediately above the apical plasma membrane and because FB28 is known to bind to chitin, chitin and its associated molecules in or near the apical ECM (adhesive zone) are likely to be the targets of FB28 and other modification inducers. Cellular adhesion to chitin may be required to propagate mechanical (physical) morphogenic signals for color pattern determination. Alternatively, but not exclusively, molecular morphogens may require chitin in the ECM to propagate themselves or to assist propagation of mechanical signals. We propose that morphogenic signals are chitin-dependent in the butterfly wing color pattern determination system.

## Figures and Tables

**Table 1:** Number of individuals with wing color pattern modifications induced by chemical injections.

Chemical	Concentration	Treated	Modified	Survived	MR(%)	SR(%)
Fluorescent brightener 28 (FB28)	25%	12	0	0	NA	0
	12.5%	13	2	2	100	15
	8.3%	6	2	2	100	33
	7.5%	29	16	16	100	55
	6.3%	3	1	1	100	33
	5.0%	9	6	9	67	100
	2.5%	18	5	16	31	89
Sodium tungstate	1.0 M	8	6	6	100	75
Heparin sodium	3.0 mg/mL	10	7	7	100	70
Dextran sulfate	10.0 mg/mL	9	9	9	100	100
Chlorfluazuron	0.0010%	7	0	7	0	100
	0.00010%	7	0	6	0	86
Captan	0.0010%	15	0	8	0	53
Congo red	10.0%	11	0	6	0	55
	1.0%	7	0	5	0	71
	0.10%	19	0	15	0	79
Polyoxin B	1.0 mg/mL	10	0	9	0	90
	0.10 mg/mL	6	0	5	0	83
Polyoxin D	1.0 mg/mL	14	0	1	0	7
	0.10 mg/mL	8	0	8	0	100
NaCl	1.0 M	10	0	10	0	100
No treatment	NA	51	0	50	0	98

The number of individuals recorded. MR: modification rate, SR: survival rate, NA: not applicable. The injection volume was 2.0 μL for all chemicals except heparin sodium (1.0 μL).

## Data Availability

Most data generated or analyzed during this study are included in this published article. If not shown, they are available upon request.
